# At the crossroads of calcium signaling, protein synthesis and neuropeptide release

**DOI:** 10.7554/eLife.106553

**Published:** 2025-04-02

**Authors:** Jakob Rupert, Dragomir Milovanovic

**Affiliations:** 1 Laboratory of Molecular Neuroscience, German Center for Neurodegenerative Diseases (DZNE) Berlin and Bonn Germany

**Keywords:** neuropeptide, exocytosis, Rab10, protein synthesis, endoplasmic reticulum, synaptic transmission, Mouse

## Abstract

By influencing calcium homeostasis, local protein synthesis and the endoplasmic reticulum, a small protein called Rab10 emerges as a crucial cytoplasmic regulator of neuropeptide secretion.

**Related research article** Dong J, Chen M, van Weering JRT, Li KW, Smit AB, Toonen RF, Verhage M. 2024. Rab10 regulates neuropeptide release by maintaining Ca2+ homeostasis and protein synthesis. *eLife* **13**:RP94930. doi: 10.7554/eLife.94930.

For a nerve signal to travel across the synapse between two neurons, the axon terminal of the first neuron – the presynaptic neuron – must release chemicals called neurotransmitters into the synaptic cleft between the neurons. These neurotransmitters then bind to receptors in the postsynaptic neuron, and the nerve signal continues on its way. However, the presynaptic neuron also releases chemicals called neuromodulators which, in simple terms, can modify how the postsynaptic neuron responds to the neurotransmitters. Examples of neuromodulators include dopamine, serotonin and small proteins called neuropeptides.

In the presynaptic regions of a neuron, neuropeptides are confined inside organelles called dense core vesicles, and several members of the Rab family of enzymes – which are all GTPases – are involved in the release of the neuropeptides from these vesicles (van den Pol, 2012; Zerial and McBride, 2001; Persoon et al., 2019). While Rab10 has the biggest influence on the release process in invertebrates (Sasidharan et al., 2012), its role in neuronal signaling in mammals is poorly understood. Now, in eLife, Matthijs Verhage (Free University of Amsterdam and the University Medical Center Amsterdam) and colleagues – including Jian Dong as first author – report the results of experiments on mouse neurons which help clarify the role of Rab10 in the release of neuropeptides (Dong et al., 2024).

One challenge when studying the involvement of Rab10 in neuropeptide release is that it also has a role in the growth of axons and dendrites. Dong et al. started by performing knock-down experiments, and confirmed that mature mouse neurons in which Rab10 was depleted had significantly shorter axons and dendrites than the control neurons with regular Rab10 levels. However, the accumulation and secretion of synaptic vesicles (inside which the neurotransmitters are confined) remained unaffected in these neurons. Furthermore, ultrastructural analysis indicated that depleting Rab10 left the overall morphology of the synapse unaffected.

Dong et al. then used a fluorescent probe to study the influence of Rab10 depletion on dense core vesicles in greater detail: the overall number of vesicles was reduced by about 30%, and the release of the vesicles from the axon terminal was reduced by about 60%. A rescue experiment in which Rab10 was overexpressed restored normal vesicle release, confirming the role of Rab10 in the process. To differentiate between the effects of Rab10 on axon/dendrite growth and vesicle release in mature neurons, Dong et al. also validated the results in neurons where Rab10 was reduced only after the initial growth.

Mass spectrometry revealed that depleting Rab10 led to a perturbation in the levels of 20% of neuronal proteins. In particular, the levels of two proteins involved in maintaining the shape of the endoplasmic reticulum – RTN4 and KDEL – were reduced. Moreover, Dong et al. confirmed that the depletion of Rab10 changed shape of the endoplasmic reticulum, as previously shown in cell lines (English and Voeltz, 2013). This is also reminiscent of the disruption of the endoplasmic reticulum that occurred when Rab10 was completely deleted in mice (Lv et al., 2015). This raises the question of how the endoplasmic reticulum is involved in neuropeptide release.

A major role of the endoplasmic reticulum in a neuron is to store and buffer calcium ions (Karagas and Venkatachalam, 2019). Dong et al. had found that SERCA2, a protein that pumps calcium ions into the endoplasmic reticulum from the cytosol, was significantly downregulated in Rab10 depleted neurons, so they used a fluorescent reporter called GCaMP to probe the concentration of calcium ions inside the neurons. They found that Rab10 depletion reduced the concentration by almost 50% in the soma, and by about 15% in the axon. Pharmacological treatments using caffeine, which triggers the release of calcium from intracellular stores, showed significantly lower cytoplasmic calcium levels in Rab10-depleted neurons and reduced the refilling of calcium ions back into the endoplasmic reticulum. Similarly, electrical stimulation of the presynaptic nerve terminals induced calcium responses that were reduced by ~20% in the depleted neurons: this indicates that the that Rab10 depletion lowers the concentration of calcium ions in the endoplasmic reticulum, slows down the refilling of this organelle with calcium ions, and decreases the calcium-dependent neuronal signaling (Figure 1).

**Figure 1.**
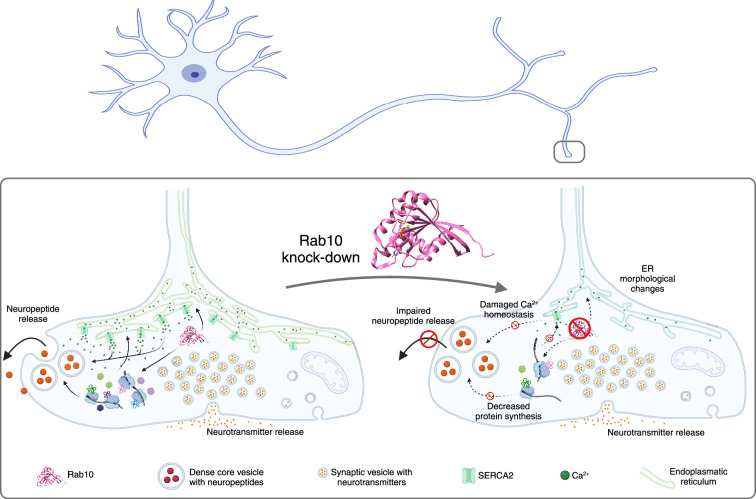
The impacts of Rab10 depletion in neurons. Top: Schematic drawing of a neuron showing the soma and dendrites on the left, and the axon and the axon terminals on the right. Lower left: The small regulatory protein Rab10 (pink) is involved in wide range of processes in an axon terminal: calcium homeostasis; maintaining the shape of the endoplasmic reticulum (ER; green); protein synthesis; and the release of neuropeptides (large orange circles). Inside the axon terminal the neuropeptides are confined inside organelles called dense core vesicles (large circular structures), and neuropeptide release involves these vesicles moving to cell membrane, fusing with it and releasing the neuropeptides. Neurotransmitters are confined inside smaller structures called synaptic vesicles before being released. Lower right: The depletion of Rab10 damages calcium homeostasis and leads to changes in the shape of the endoplasmic reticulum. Rab10 depletion also results in a reduction in overall protein synthesis, which disrupts the release of neuropeptides, but not the release of neurotransmitters.

Created with BioRender.com using data from Rai et al., 2024.

Dong et al. then employed a different approach – using a calcium ionophore to trigger an influx of extracellular calcium ions – to stimulate the release of neuropeptides. The number of dense core vesicles was similar for the control and the Rab10-depleted neurons, but the number of vesicles that fused with the membrane of the neuron was reduced. This strongly suggests that calcium has some role in the mechanisms by which Rab10 influences neuropeptide release. However, the fact that this reduction was smaller than the reduction observed in the electrical stimulation experiments indicates that factors besides calcium ions also have a role. This could explain why overexpression of the SERCA2 calcium pump in Rab10-depleted neurons failed to rescue the release of the dense core vesicles.

To explore further, Dong et al. sought to rescue the decreased protein synthesis seen in Rab10-depleted neurons by adding the essential amino acid leucine. Although the addition of leucine did not alter the changes to the shape of the endoplasmic reticulum, it did lead to an increase in the release of dense core vesicles. Thus, the effect of Rab10 on neurotransmission is likely due to a combination of its direct effect on the release mechanisms of dense core vesicles and indirect effects via its role in regulating the synthesis of proteins involved in the dynamics of the endoplasmic reticulum and the growth of axons and dendrites (Figure 1).

Given the dense packing of organelles and biomolecular condensates within axon terminals (Sansevrino et al., 2023), where local translation seems to have an important role (Hafner et al., 2019; Rankovic et al., 2025), this study points to an exciting potential role for Rab10. This small protein selectively regulates the neuropeptide release through its ability to influence the morphology of the endoplasmic reticulum, calcium signaling and local protein translation at the synapse. Such selectivity would allow for tailored interventions to treat neurodevelopmental and psychiatric diseases caused by the disruption of Rab10 signaling and neuropeptide release.
